# A Network Pharmacology to Explore the Mechanism of *Astragalus* Membranaceus in the Treatment of Diabetic Retinopathy

**DOI:** 10.1155/2020/8878569

**Published:** 2020-11-02

**Authors:** Qi Jin, Xiao-Feng Hao, Li-Ke Xie, Jing Xu, Mei Sun, Hang Yuan, Shi-Hui Wang, Gai-Ping Wu, Meng-Lu Miao

**Affiliations:** Surgical Department of Fundus Disease and Trauma, Eye Hospital, China Academy of Chinese Medical Sciences, Beijing 100040, China

## Abstract

**Background:**

Diabetic retinopathy (DR) includes a series of typical lesions affected by retinal microvascular damage caused by diabetes mellitus (DM), which not only seriously damages the vision, affecting the life's quality of patients, but also brings a considerable burden to the family and society. *Astragalus* Membranaceus (AM) is a commonly used medicine in clinical therapy of eye disorders in traditional Chinese medicine (TCM). In recent years, it is also used for treating DR, but the specific mechanism is unclear. Therefore, this study explores the potential mechanism of AM in DR treatment by using network pharmacology.

**Methods:**

Based on the oral bioavailability (OB) and drug likeness (DL) of two ADME (absorption, distribution, metabolism, excretion) parameters, Traditional Chinese Medicine Systems Pharmacology Database (TCMSP), Swiss Target Prediction platform, GeneCards, and OMIM database were used to predict and screen the active compounds of AM, the core targets of AM in DR treatment. The Metascape data platform was used to perform Gene Ontology (GO) and Kyoto Encyclopedia of Genes and Genomes (KEGG) pathway enrichment analysis on the core targets.

**Results:**

24 active compounds were obtained, such as quercetin, kaempferol, and astragaloside IV. There were 169 effective targets of AM in DR treatment, and the targets were further screened and finally, 38 core targets were obtained, such as VEGFA, AKT1, and IL-6. EGFR tyrosine kinase inhibitor resistance, AGE-RAGE signaling pathway in diabetic complications, PI3K-Akt signaling pathway, and other metabolic pathways participated in oxidative stress, cell apoptosis, angiogenesis signal transduction, inflammation, and other biological processes.

**Conclusion:**

AM treats DR through multiple compounds, multiple targets, and multiple pathways. AM may play a role in the treatment of DR by targeting VEGFA, AKT1, and IL-6 and participating in oxidative stress, angiogenesis, and inflammation.

## 1. Introduction

Diabetes mellitus (DM) is a metabolic disorder caused by genetic and environment factors, and it has become a critical health problem worldwide due to its high prevalence and related disability and mortality [1]. In a recent cross-sectional study, it was observed that the prevalence of diabetes had increased slightly among adults living in China from 2007 to 2017 [2]. Paralleling with the increasing pandemic of DM, diabetic retinopathy (DR) is one of the complications of diabetes. The prevalence of DR is also growing, and an epidemiological study from urban Chinese indicated that the occurrence of DR was 8.1% for patients with DM [3]. DR destroyed the normal function of the retina and was a threat for sight in adults aged 20–74 years [4], which seriously affects the quality of patients' life [5]. Moreover, DR needs repeated treatment, which brings heavy financial burden to the family and society [6]. Therefore, early diagnosis and early treatment are particularly important. Clinically, most conventional treatments such as maintaining blood glucose and lipids and keeping a healthy lifestyle are adopted. However, there are certain limitations, so how to effectively prevent and treat DR has become a current research hotspot [7].

Recently, traditional Chinese medicine (TCM) is gradually attracting public's attention owing to its clinical efficacy. Compared with western medicine, TCM is an important alternative strategy because of syndrome differentiation and holistic concept for treating DM [8]. Also, there are various treatment principles for DR, including boosting qi and nourishing yin, fortifying the spleen and removing dampness, invigorating blood, and unblocking the collaterals [9]. Through treatment according to pattern differentiation, herbal medicines have certain advantages in preventing and treating DR [10].

*Astragalus* Membranaceus (AM) is named “*Huangqi*” of TCM. AM was originally described in the Sheng Nong's Herbal Classic which is the Classic of Herbal Medicine in ancient TCM practice, which is the dry root of astragalus mongolicus or membranous astragalus [11]. It is one of the popular health-promoting herbs that have been used to strengthen immunity of people for more than 2000 years. Furthermore, it contains numerous constituents, such as saponins, flavonoids, and polysaccharides, which have wide biological activities, for example, anti-inflammatory, antithrombotic, antioxidant, and antidiabetic activities. Chinese herbs have been used since centuries for the treatment and prevention of various disorders. There were studies that AM was found upregulating insulin-signaling pathways by improving the activity of casein kinase, regulating lipid metabolism, and enhancing insulin resistance to treat T2DM [12]. Owing to properties that they may alleviate several hyperglycaemia-induced pathological occurrences in the retina, AM can potentially be used for the treatment and prevention of DR [13]. There were numerous studies which reported that AM could downregulate the signaling pathway of p38 MAPK, simultaneously inhibit the mediated pathway of NF-*κ*B, and reduce the production of proinflammatory cytokines, such as IL-1*β* and TNF-*α*, which were induced by AGE. Moreover, it can inhibit the formation of retinal angiogenesis by downregulating the level of VEGF in Muller cells induced by hyperglycemia [14].

**Figure 1:**
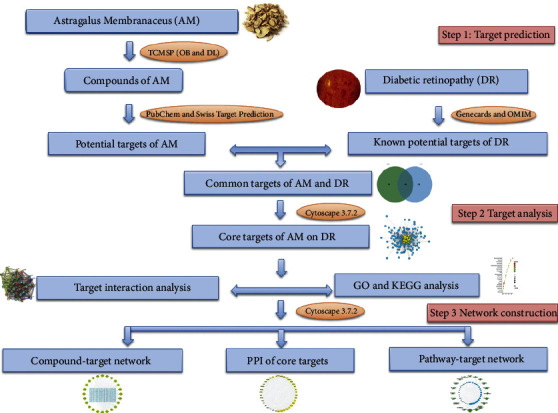
Flow chart of the study. DR: diabetic retinopathy; AM: *Astragalus* Membranaceus; TCMSP: Traditional Chinese Medicine Systems Pharmacology Database; GO: Gene Ontology; KEGG: Kyoto Encyclopedia of Genes and Genomes; PPI: protein-protein interaction.

However, the specific mechanism of action is not clear. In recent years, a new trend emerged by using network pharmacology to understand TCM. From the perspective of systems biology, network pharmacology explores the relationship between drugs-components-targets-diseases, predicts the potential mechanism of drugs to treat diseases, and provides a theoretical basis for effectiveness of TCM to treat the disease [15]. Therefore, this study used the method of network pharmacology, collecting data and information from multiple data platforms and exploring the potential mechanism of AM in DR treatment, to provide a theoretical basis for the prevention and treatment of DR by AM. The overall flow chart of the study is shown in Figure 1.

## 2. Materials and Methods

### 2.1. Screening of Potential Compounds and Targets of the Herb

“Huangqi” (AM) was retrieved in TCMSP and screened with OB ≥ 30% and DL ≥ 0.18. The chemical compounds of AM were obtained, and the compounds of AM from the literature reports were added. All the components of AM were combined. The PubChem platform (https://pubchem.ncbi.nlm.nih.gov/, 2020.6.04) platform was used, and the InCHIKey number or CAS number of the effective components was entered in order to obtain the 2D structural formula of the components. And then, the 2D structural formula was imported into the Swiss Target Prediction platform (http://www.swisstargetprediction.ch/), 2020.6.04 [16]. The species was selected to be “*Homo sapiens*,” and the potential targets of the compounds with the condition of “probability ≥  0” were predicted. Finally, targets obtained from both databases were combined and deduplicated, and the targets of AM were obtained.

TCMSP: The Traditional Chinese Medicine Systems Pharmacology Database and Analysis Platform (TCMSP) (https://tcmspw.com/tcmsp.php) is a database that is based on the framework of systems pharmacology for herbal medicine, which can predict the compound and target of herbal medicines by setting ADME (absorption, distribution, metabolism, excretion) parameters [17].

ADME parameters: Most herbal medicines are mainly taken orally and undergo metabolic processes, such as metabolism, absorption, distribution, and excretion (ADME) in the human body, including OB and DL.

Oral bioavailability (OB): OB refers to the speed and degree of absorption of the active compounds or active groups of the drug in the systemic circulation, and is a key parameter for evaluating whether the drug can be developed. Generally, molecules with OB ≥ 30% have good oral availability [18].

Drug likeness (DL): the DL of all herbal medicines is acquired by calculation of Tanimoto coefficient. The Tanimoto coefficient is defined as follows [19]:

(1) $$\begin{array}{c} f\left(A,B\right)=\frac{A\cdot B}{{\left|A\right|}^{2}+{\left|B\right|}^{2}-A\cdot B}. \end{array}$$

Here, *A* is the molecular descriptor index of the compounds of herbal medicines to be tested in the TCMSP database and *B* is the average drug likeness index described by all 6511 small drug molecules in the DrugBank database. The descriptors of all molecules are calculated by the Dragon software (http://www.talete.mi.it/index.htm). Generally, molecules with DL ≥ 0.18 have good drug likeness.

PubChem: PubChem is the world's largest collection platform of chemical information and search information of compounds, such as chemical and physical properties, biological activities, safety, and toxicity information and more and information can be obtained by importing name, molecular formula, structure, and other identifiers of compounds [20].

Swiss Target Prediction: Swiss Target Prediction is a type of online analysis software for predicting targets of small molecules, which can import SMILES and structure of the molecule to predict the targets of small molecule compounds [21].

### 2.2. Screening of Potential Targets of the Disease

The keyword “diabetic retinopathy” was searched in the GeneCards Database (https://www.genecards.org/, 2020.6.05) [22], and the related targets of DR were obtained. In the OMIM database (https://omim.org/,2020.6.05) [23], Gene Map was selected in “Advanced Search” and then the keyword “diabetic retinopathy” was entered, and the related targets of DR were obtained. The targets obtained from the two databases were integrated and duplicated, and the targets of disease were obtained.

Online Mendelian Inheritance in Man (OMIM) and GeneCards are two different databases that provide comprehensive information of all annotated and predicted human genes.

### 2.3. Screening of Core Targets of AM in DR Treatment

The jvenn online platform (http://jvenn.toulouse.inra.fr/app/example.html) [24] was used for core targets. The targets of the drug and disease were, respectively, imported, and the intersection targets between drug and disease were obtained. Then, the String platform (https://string-db.org/) was used and protein-protein interaction (PPI) was built. In the platform, “Multiple proteins” was selected and the organism was selected as “*Homo sapiens*.” The intersection targets of AM and DR were then imported, the medium confidence of minimum required interaction score was set as 0.4, and the remaining parameters were maintained at the default settings [25]. The file was exported in “TSV format,” and then, Cytoscape 3.7.2 was used to analyze the topological properties of the PPI network. The degree values were calculated, and the core targets of AM in DR treatment with degree ≥ 2 × median were selected.

STRING: it is a search tool for the retrieval of interacting genes/proteins, which is applied for predicting the PPI network and detecting the possible relationships.

Cytoscape: it is a type of open-source software used to analyze and visualize biological networks [26].

### 2.4. GO and KEGG Pathway Enrichment Analysis

The core targets of AM in DR treatment were imported into the Metascape platform, and in this, species was selected as “H sapiens,” “Custom analysis” was selected, and parameters of *P* value < 0.01, a minimum count of 3, and an enrichment factor >1.5 (the enrichment factor is the ratio between the observed counts and the counts expected by chance) were set. GO and KEGG pathway enrichment analyses on core targets were performed.

Metascape: it is a gene function annotation analysis tool, to realize the recognition of gene or protein function through the bioinformatics analysis of genes and proteins [27].

Gene Ontology (GO): it is a bioinformatics tool used for annotating genes, gene products, and sequences to analyze their biological processes. GO includes 3 aspects: molecular function (MF); biological process (BP); cellular component (CC).

KEGG: it is a database resource comprising information on the biological systems of genome sequences and other high-throughput data [28].

### 2.5. Construction of Networks

#### 2.5.1. Compound-Target Network

Cytoscape 3.7.2 was used to construct the component-target network (C-T network).

#### 2.5.2. PPI of Core Targets

String online platform was used to build PPI of core targets, and then, PPI was visualized by Cytoscape 3.7.2.

#### 2.5.3. Target-Pathway Network

The top 20 KEGG pathways were screened, and Cytoscape 3.7.2 was used to construct the target-pathway network (T-P network).

## 3. Results

### 3.1. Active Compounds and Targets of the Drug

On the TCMSP platform, the ADME parameters of OB ≥ 30% and DL ≥ 0.18 were selected 20 active compounds were obtained, and then, according to the relevant literature [29], additional 4 active compounds were obtained and merged with the former, and finally, a total of 24 compounds of AM were identified (Table 1).

**Figure 2:**
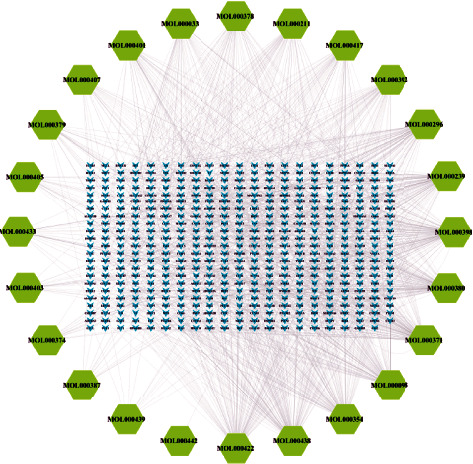
C-T network. The layout of the outer ring and the inner rectangular were constructed according to different nodes, and the green nodes represent the compound while blue represents the target; the edges among the nodes represent the relationship between the compound and the targets. The more the edges of nodes, the more important the nodes.

2D structural formulas of 24 compounds were obtained from PubChem, and then, compounds were imported into the Swiss Target Prediction platform to obtain the target information of 24 compounds. Targets were merged and deduplicated, and a total of 482 targets of AM were obtained. The component-target network (C-T network) was constructed (Figure 2) by using Cytoscape 3.7.2, and there were 506 nodes and 1310 edges. The function of “Network Analyzer” in Cytoscape 3.7.2 was used to perform topological analysis on the components (Table 2), and the main compounds were jaranol, isoflavanone, kaempferol, and quercetin.

### 3.2. Core Targets of AM on DR

**Figure 3:**
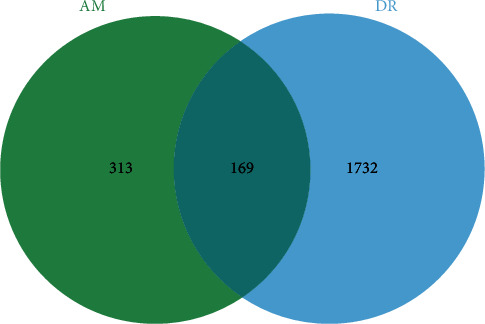
Common targets of AM and DR. There were 482 targets of AM (left), while 1901 targets of DR (right), and 169 common targets between AM and DR (middle).

**Figure 4:**
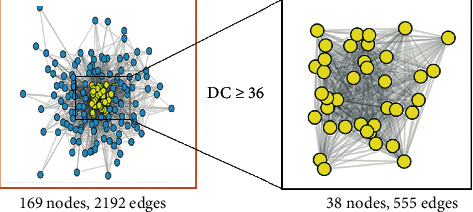
The process of screening core targets. From 169 common targets (left), selection of 38 core targets with degree ≥ 36 (right).

There were 217 targets of DR in the OMIM database, while there were 1753 targets of DR in the GeneCards database. The targets from the two databases were merged, the duplicates were removed, and finally, 1901 targets of DR were obtained. 482 targets of AM and 1901 targets of DR were imported into Venny2.1.0 online platform, and there were 169 common targets (Figure 3). 169 targets were imported into STRING online platform, and a file in “TSV” format was exported. Then, it was imported into Cytoscape 3.7.2, the function of “network analyzer” was used, and the median of degree was set as 18. 38 core targets of AM in DR treatment with degree ≥ 36 (2 × 18) were screened (Figure 4).

### 3.3. PPI of Core Targets

**Figure 5:**
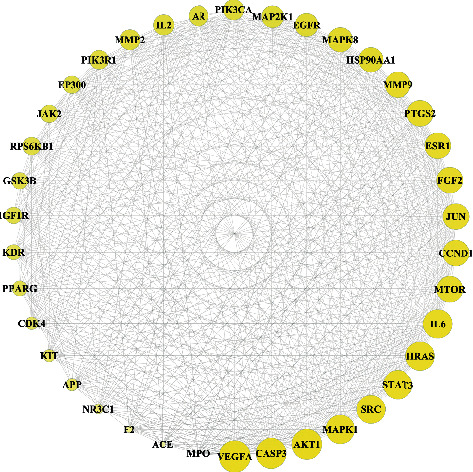
PPI of core targets. The layout of the outer ring went in an anticlockwise direction according to the area and color of the nodes, andthe yellow nodes represent the core targets of AM on DR; the size and transparency of the nodes were divided according to the degree value from large to small. The inner edges represent the relationship between the core targets. The more the edges of the nodes were, the larger the area of the nodes, the darker the color, and the more important the targets.

38 core targets were imported into the STRING platform, the PPI of the core targets were obtained (Figure 5), and finally, there were 38 nodes and 555 edges. The average node degree was 29.2. The specific information of targets is shown in Table 3.

### 3.4. Enrichment Analysis of 38 Targets

There were 38 targets of AM on DR; the biological function of the 38 targets was elucidated. GO analysis and KEGG pathway analysis on 38 targets were performed. Based on GO results, there were 79 processes of molecular functions, such as protein kinase activity, protein serine/threonine kinase activity, and kinase binding; there were 418 biological processes, such as transmembrane receptor protein tyrosine kinase signaling pathway, positive regulation of cell migration, and positive regulation of cell motility; there were 42 cellular components, such as membrane raft, membrane microdomain, and membrane region. In addition, there were 123 pathways for results of KEGG.

**Figure 6:**
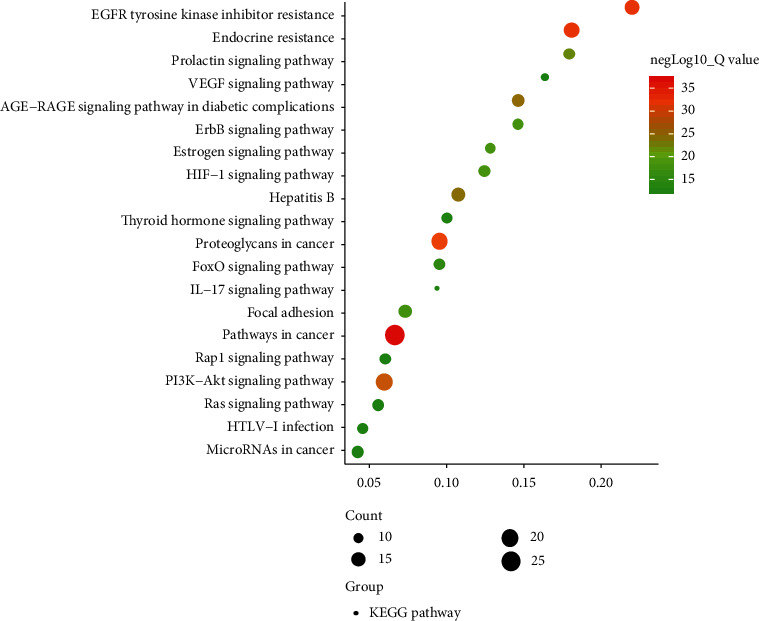
The top 20 results of KEGG pathway. The larger the area, the more the count, the redder the color, and the smaller the *P*value.

**Figure 7:**
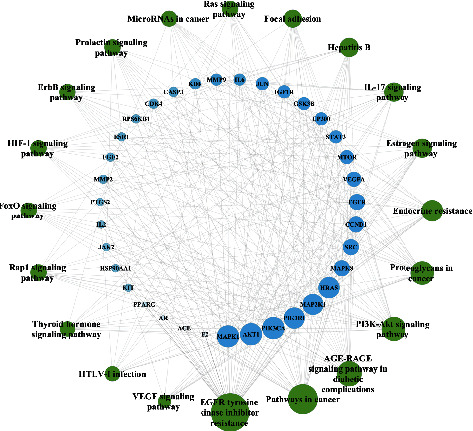
T-P network. The layout of the outer and inner ring went in an anticlockwise direction according to the area and color of the nodes; the green nodes represent the pathways while the blue nodes represent targets. The size and transparency of the nodes were divided according to the degree value from large to small. The inner edges represented the relationship between the pathways and the targets. The more edges, the larger the area of the nodes, the darker of the color, and the more important the nodes.

The top 20 results of KEGG were listed and the scatter plot was drawn. The larger the dot, the more the count, the redder the color, and the smaller the *P* value (Figure 6), and EGFR tyrosine kinase inhibitor resistance, AGE-RAGE signaling pathway in diabetic complications, PI3K-AKT signaling pathway and IL-17 signaling pathway may be involved in the treatment of DR by AM. The top 20 pathways were screened and the target-pathway network (T-P network) was constructed (Figure 7). There were 55 nodes and 331 edges, and the topology analysis was further analyzed. The main pathways included EGFR tyrosine kinase inhibitor resistance, AGE-RAGE signaling pathway in diabetic complications, and PI3K-Akt signaling pathway (Table 4).

## 4. Discussion

DR is a common and severe complication of DM, which is the main cause of blindness in adults. In the early stage, the high-glucose environment influences the retinal microvascular damage, which is characterized by loss of retinal cells, thickening of the basement membrane of capillaries, expansion and deformation of capillaries, and destruction of the blood-retinal barrier. Moreover, the progression is characterized by the occlusion of capillaries and the formation of pathological neovascularization, retinal hemorrhage, etc, which cause irreversible loss of vision [30]. Experts and scholars have been devoted to the research of effective prevention and treatment of DR, but there are still many unsolved mysteries. In recent years, studies reported that the treatment of this disease by TCM had been increasing year by year and the treatment efficiency had gradually improved. AM is a class of herbs used for boosting qi and invigorating blood, but its precise mechanism of AM on DR is not yet clear. Therefore, it is necessary to explore the mechanism of AM in DR treatment by using functions of screening compounds, predicting targets, and analyzing pathways of drugs in network pharmacology.

In this study, we firstly identified the active compounds of AM and combined literature reviews. 24 compounds were identified, and the specific information of compounds is shown in Table 1. Some compounds, such as quercetin, kaempferol, and astragaloside IV were reported to have played an important role in DR. Quercetin could reduce the expression of MMP-9 and VEGF in rats with diabetic retinopathy [31] and inhibit the generation of angiogenesis both in vitro and ex vivo [32]. Quercetin can reduce the overproduction of MCP, IL-6, and ROS and downregulate the expression of P53, Bax, and Caspase-3 by promoting PTEN/AKT pathway and inhibiting NF-*κ*B pathway, which could protect ARPE-19 cell damage induced by high glucose [33]. Kaempferol could protect against oxidative stress leading to damage of human RPE cells through its antioxidant activity and antiapoptosis function [34]. Moreover, it could suppress angiogenesis of human retinal endothelial cells (HRECs) via targeting VEGF and PGF to inhibit the activation of Src-Akt1-Erk1/2 signaling pathway under glucose conditions [35]. Astragaloside IV could prevent the activation of ERK1/2 phosphorylation and NF-*κ*B and further relieve the RGCs dysfunction in ab/db mice with DR [36], and it has potential protective effects on retinal capillary endothelial cells (RCECs) incubated with high glucose conditions [37].

We predicated 169 common targets between AM and DR, and on further screening, 38 core targets for AM in DR treatment were obtained. The targets were mainly enriched for oxidative stress, cell proliferation, apoptosis, inflammation, etc. When it comes to core targets, Akt1, VEGFA, IL-6, Caspase-3, and STAT3 played an important role in the process of AM in DR treatment. The detailed information is given in Table 2. VEGFA could be an activator of the stimulation of angiogenesis, and particularly, it was reported to be strongly upregulated in PDR [38]. Numerous studies had reported that AKT signaling pathway could be involved in the occurrence and development of DR and the activation of AKT can maintain the steady state of endothelial function of retinal capillaries [39]. Caspase-3 could participate in the pathological process of DR by regulating apoptosis, and the abnormally high expression level of Caspase-3 may be correlated with the severity of DR [40]. The inhibition of STAT13 can alleviate retinal inflammation and reduce retinal cell death in rats of DR, while IL-6 could also be a proinflammatory factor, and and by inhibiting the activation of IL-6/STAT3 signaling pathway and then affecting the oxidation and antioxidation process of the retina, the purpose of protecting the retina of diabetic rats can be achieved [41].

**Figure 8:**
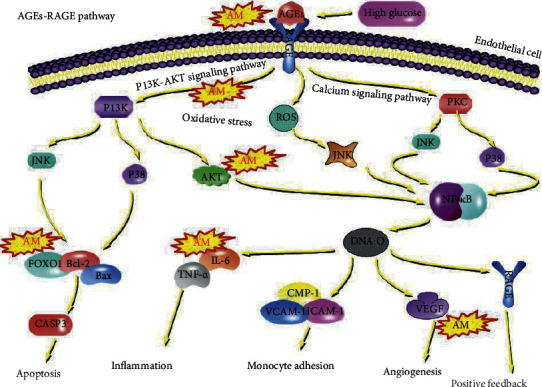
AGEs-RAGE pathway. Cited from the KEGG database (https://www.kegg.jp/). The position of the yellow icon indicates that AM maybe act as a key node of the pathway.

Subsequently, we compared the results of GO and KEGG enrichment analyses for 38 key targets, and the results indicated that regulation of various kinase activities, response to insulin, and regulation of vascular endothelial cells played a main role in the process of AM in DR treatment. Furthermore, in delaying the progression of diabetic retinopathy, oxidative stress and inflammation could also play an important role [42]. The action pathway mainly includes EGFR tyrosine kinase inhibitor resistance, AGE-RAGE signaling pathway in diabetic complications, and PI3K-Akt signaling pathways. Previous studies had indicated that renal epidermal growth factor receptors (EGFRs) were activated in models of diabetic nephropathy (DN), and inhibition of EGFR activity protected against progressive DN in T1 DM and T2 DM [43]. So the EGFR tyrosine kinase inhibitor resistance may delay the progression of diabetes and improve complications such as diabetic retinopathy. In the condition of high glucose, nonenzymatic glycosylation of proteins and lipids can be induced, advanced glycation end-products (AGEs) can be generated, and through the activation of AGE receptor (RAGE), oxidative stress and inflammation can be induced [44], which could damage the retina. The details of AM on the AGEs-RAGE pathway are shown in Figure 8, and it was cited from the KEGG database (https://www.kegg.jp/).

In addition, there were several pathways, such as pathway in cancer, proteoglycans in cancer, and hepatitis bare, indicating AM might exhibit potential therapeutic effects on these diseases, while demonstrating that the progression of DR is influenced by other diseases. There were multiple targets, multiple pathways, and multiple processes involved in treatment of DR through AM. In addition, there were still some deficiencies in this study. The network pharmacology method was used to predict the targets and pathways of AM in DR treatment, which still need to be verified by future pharmacological studies.

## 5. Conclusions

In summary, we found that AM in DR treatment has the characteristics of multiple components, multiple targets, and multiple pathways through network pharmacology. AGE-RAGE signaling pathway in diabetic complications and PI3K-Akt signaling pathway might play an important role in the treatment process, providing a theoretical basis for AM in DR treatment and a new direction for the development of drugs for DR.

## Figures and Tables

**Table 1:** Information of compounds from AM.

Number	Mol ID	Molecule name	Molecule weight	OB (%)	DL
1	MOL000211	Mairin	456.78	55.38	0.78
2	MOL000239	Jaranol	314.31	50.83	0.29
3	MOL000296	Hederagenin	414.79	36.91	0.75
4	MOL000033	(3S,8S,9S,10R,13R,14S,17R)-10,13-dimethyl-17-[(2R,5S)-5-propan-2-yloctan-2-yl]-2,3,4,7,8,9,11,12,14,15,16,17-dodecahydro-1H-cyclopenta (a) phenanthren-3-ol	428.82	36.23	0.78
5	MOL000354	Isorhamnetin	316.28	49.6	0.31
6	MOL000371	3,9-di-O-Methylnissolin	314.36	53.74	0.48
7	MOL000374	5′-Hydroxyiso-muronulatol-2′,5′-di-O-glucoside	642.67	41.72	0.69
8	MOL000378	7-O-Methylisomucronulatol	316.38	74.69	0.3
9	MOL000379	9,10-Dimethoxypterocarpan-3-O-*β*-D-glucoside	462.49	36.74	0.92
10	MOL000380	(6aR,11aR)-9,10-Dimethoxy-6a,11a-dihydro-6H-benzofurano (3,2-c) chromen-3-ol	300.33	64.26	0.42
11	MOL000387	Bifendate	418.38	31.1	0.67
12	MOL000392	Formononetin	268.28	69.67	0.21
13	MOL000398	Isoflavanone	316.33	109.99	0.3
14	MOL000417	Calycosin	284.28	47.75	0.24
15	MOL000422	Kaempferol	286.25	41.88	0.24
16	MOL000433	FA	441.45	68.96	0.71
17	MOL000438	(3R)-3-(2-Hydroxy-3,4-dimethoxyphenyl)chroman-7-ol	302.35	67.67	0.26
18	MOL000439	Isomucronulatol-7,2′-di-O-glucosiole	626.67	49.28	0.62
19	MOL000442	1,7-Dihydroxy-3,9-dimethoxy pterocarpene	314.31	39.05	0.48
20	MOL000098	Quercetin	302.25	46.43	0.28
21	MOL000401	Astragaloside I	869.17	46.79	0.11
22	MOL000403	Astragaloside II	827.13	46.06	0.13
23	MOL000405	Astragaloside III	785.09	31.83	0.1
24	MOL000407	Astragaloside IV	785.09	22.5	0.15

**Table 2:** Key compounds of AM.

Mol ID	Name	DC	CC	BC
MOL000422	Kaempferol	100	0.390867	0.078562
MOL000354	Isorhamnetin	100	0.390867	0.070723
MOL000098	Quercetin	100	0.390867	0.070715
MOL000239	Jaranol	100	0.390263	0.09292
MOL000438	(3R)-3-(2-Hydroxy-3,4-dimethoxyphenyl)chroman-7-ol	100	0.38966	0.14801
MOL000398	Isoflavanone	100	0.38906	0.212952
MOL000371	3,9-di-O-Methylnissolin	100	0.38727	0.212242
MOL000380	(6aR,11aR)-9,10-Dimethoxy-6a,11a-dihydro-6H-benzofurano (3,2-c)chromen-3-ol	100	0.386677	0.13606

DC = degree centrality; CC = closeness centrality; BC = betweenness centrality.

**Table 3:** Specific information for core targets.

Number	Uniprot ID	Gene name	Protein name	Counts
1	P31749	AKT1	RAC-alpha serine/threonine-protein kinase	36
2	P15692	VEGFA	Vascular endothelial growth factor A	37
3	P05231	IL6	Interleukin-6	35
4	P42574	CASP3	Caspase-3	36
5	P12931	SRC	Proto-oncogene tyrosine-protein kinase Src	35
6	P00533	EGFR	Epidermal growth factor receptor	31
7	P28482	MAPK1	Mitogen-activated protein kinase 1	35
8	P40763	STAT3	Signal transducer and activator of transcription 3	35
9	P05412	JUN	Transcription factor AP-1	33
10	P01112	HRAS	GTPase HRas	35
11	P07900	HSP90AA1	Heat shock protein HSP 90-alpha	32
12	P45983	MAPK8	Mitogen-activated protein kinase 8	32
13	P42345	MTOR	Serine/threonine-protein kinase mTOR	33
14	P24385	CCND1	G1/S-specific cyclin-D1	33
15	P03372	ESR1	Estrogen receptor	33
16	P09038	FGF2	Fibroblast growth factor 2	33
17	P14780	MMP9	Matrix metalloproteinase-9	33
18	P35354	PTGS2	Prostaglandin G/H synthase 2	33
19	P37231	PPARG	Peroxisome proliferator-activated receptor gamma	25
20	P42336	PIK3CA	Phosphatidylinositol 4,5-bisphosphate 3-kinase catalytic subunit alpha isoform	29
21	P10275	AR	Androgen receptor	29
22	P08253	MMP2	72 kDa type IV collagenase	29
23	P35968	KDR	Vascular endothelial growth factor receptor 2	25
24	Q09472	EP300	Histone acetyltransferase p300	27
25	P27986	PIK3R1	Phosphatidylinositol 3-kinase regulatory subunit alpha	28
26	P60568	IL2	Interleukin-2	29
27	P12821	ACE	Angiotensin-converting enzyme	18
28	O60674	JAK2	Tyrosine-protein kinase JAK2	27
29	P05067	APP	Amyloid-beta precursor protein	23
30	Q02750	MAP2K1	Dual specificity mitogen-activated protein kinase kinase 1	30
31	P08069	IGF1R	Insulin-like growth factor 1 receptor	26
32	P05164	MPO	Myeloperoxidase	16
33	P23443	RPS6KB1	Ribosomal protein S6 kinase beta-1	27
34	P04150	NR3C1	Glucocorticoid receptor	21
35	P10721	KIT	Mast/stem cell growth factor receptor kit	23
36	P49841	GSK3B	Glycogen synthase kinase-3 beta	26
37	P11802	CDK4	Cyclin-dependent kinase 4	23
38	P00734	F2	Prothrombin	19

**Table 4:** The main pathway of KEGG.

Term	Name	BC	CC	DC
hsa01521	EGFR tyrosine kinase inhibitor resistance	0.23113	0.739726	35
hsa05200	Pathways in cancer	0.094192	0.606742	27
hsa04933	AGE-RAGE signaling pathway in diabetic complications	0.044391	0.545455	22
hsa04151	PI3K-Akt signaling pathway	0.049164	0.534653	21
hsa05205	Proteoglycans in cancer	0.036825	0.524272	20

DC = degree centrality; CC = closeness centrality; BC = betweenness centrality.

## Data Availability

The data of this study are available from the corresponding author upon reasonable request.
